# Integrative genomic analysis reveals a conserved role for prolactin signalling in the regulation of adrenal function

**DOI:** 10.1002/ctm2.630

**Published:** 2021-11-08

**Authors:** Carmen Ruggiero, Barbara Altieri, Edith Arnold, Lourdes Siqueiros‐Marquez, Mabrouka Doghman‐Bouguerra, Mario Detomas, Nelly Durand, Marielle Jarjat, Max Kurlbaum, Fabrice Chatonnet, Timo Deutschbein, Carmen Clapp, Enzo Lalli

**Affiliations:** ^1^ Institut de Pharmacologie Moléculaire et Cellulaire CNRS UMR 7275 Valbonne France; ^2^ Université Côte d'Azur Valbonne France; ^3^ Department of Internal Medicine I–Division of Endocrinology and Diabetes University Hospital University of Würzburg Würzburg Germany; ^4^ Instituto de Neurobiología Universidad Nacional Autónoma de México (UNAM) Queretaro Mexico; ^5^ CONACYT‐Instituto de Neurobiología Universidad Nacional Autónoma de México (UNAM) Queretaro Mexico; ^6^ Central Laboratory Core Unit Clinical Mass Spectrometry University Hospital Würzburg Würzburg Germany; ^7^ Université de Rennes 1 Inserm Établissement Français du Sang de Bretagne Rennes France; ^8^ Laboratoire d'Hématologie Pôle de Biologie Centre Hospitalier Universitaire Rennes France; ^9^ Medicover Oldenburg MVZ Oldenburg Germany; ^10^ Inserm Valbonne France

Dear Editor,

We report here that the pituitary hormone prolactin (PRL) has an important, conserved role in regulating adrenal gland function.

The adrenal plays a pivotal role in endocrine homeostasis and stress response, which vary across lifespan and have different features in the two sexes.^[^ ^1^ ^]^ Furthermore, most adrenal disorders have a higher prevalence in women.^[^ ^2^ ^]^ To get deeper insight into the mechanisms of age‐ and sex‐dependent adrenal function, we performed an integrative analysis of the mouse adrenal transcriptome by RNA‐seq and active enhancer (as defined by H3K27ac ChIP‐seq) usage at different ages (from E18.5 to P12 weeks) in both sexes. Multivariate analysis showed that age, but not sex, had a significant effect on adrenal global gene expression profiles (Figure  and Supporting information S1; Tables S1, S2). Expression of genes related to lipid metabolism and immunity was progressively enriched with age (Figure 1C and Supporting information Table S3), with the proportion of macrophages increasing and neutrophils/mast cells decreasing at older ages (Figure 1D). The expression of different classes of secreted proteins, ion channels and enzymes showed a lower level of sex‐dependent enrichment (Figure 1E and Supporting information Table S4). Age‐and sex‐dependent long non‐coding RNA differentially expressed genes (DEG) followed the same pattern as coding DEG, being mostly specific for each age and sex (Supporting information Figure S2 and Table S5). Remarkably, P12 weeks adrenals of both sexes showed increased percentages of novel gene transcripts compared to previous ages (Supporting information Figure S2E). Sexually dimorphic expression pervades all cell populations of the mouse adrenal gland (Supporting information Figure S3 and Table S6).

**FIGURE 1:**
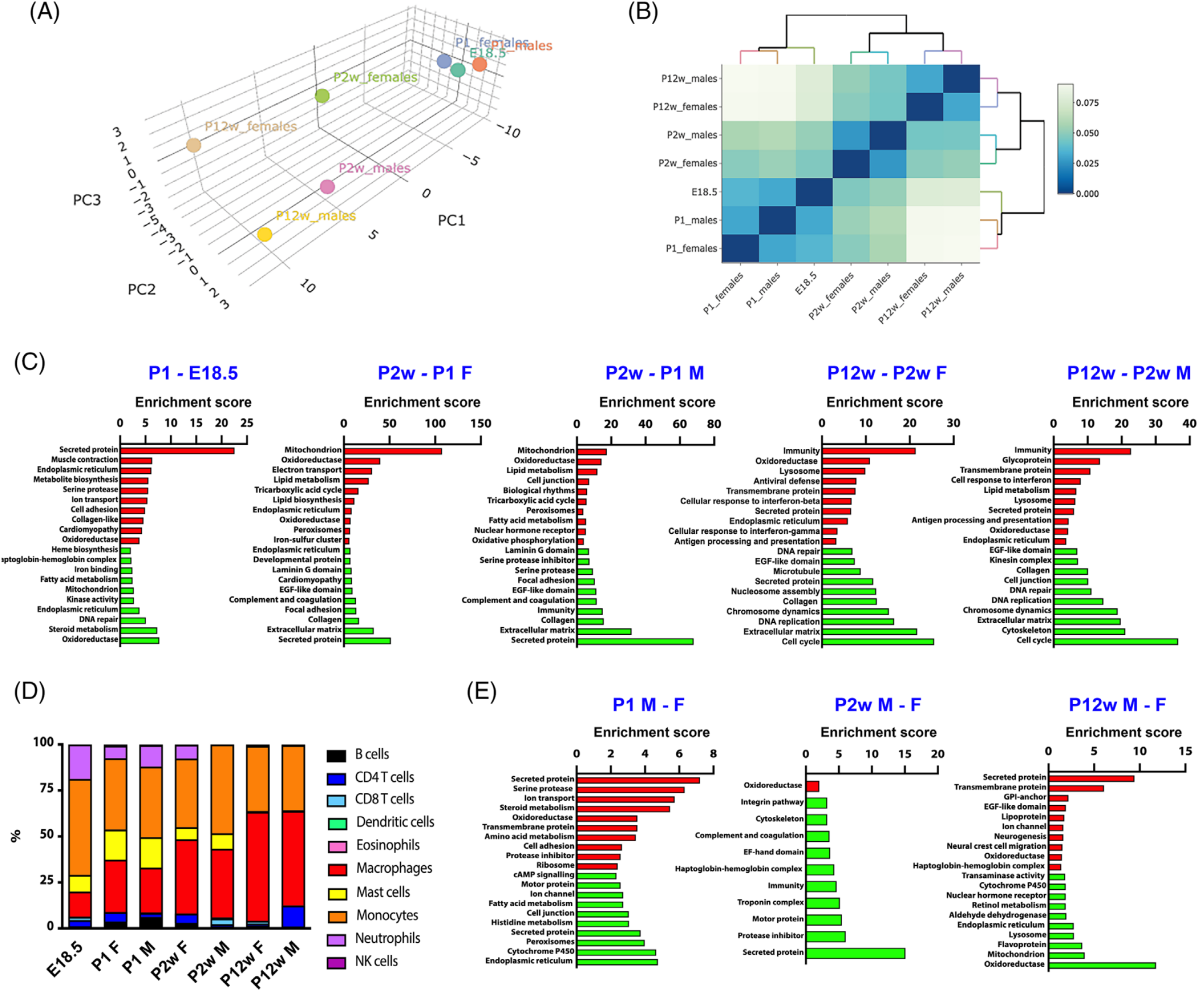
Age‐ and sex‐dependent gene expression profiles in the mouse adrenal gland. (A) Principal component analysis (PCA) plot of global gene expression at E18.5 and P1, P2 weeks and P12 weeks adrenals in both sexes. Proportions of variance: PC1, 85.46%; PC2, 7.23%; PC3, 4.11%. Effect of: age, *p *= 0.0083; sex, *p *= 0.4338; age‐sex interaction, *p *= 0.2896. Permutational multivariate analysis (PERMANOVA). (B) Dendrogram plot of hierarchical sample clustering of the different adrenal samples. Distance among clusters is indicated with a colour scale. (C) GO classification of mouse adrenal gland DEG according to age. Red: upregulated GO categories in older compared to younger animals at each comparison. Green: downregulated GO categories in older compared to younger animals at each comparison. (D) Relative percentages of Immune cell populations infiltrating the mouse adrenal gland at each age and for each sex. (E) GO classification of mouse adrenal gland DEG according to sex. Red: upregulated GO categories in males compared to females at each comparison. Green: downregulated GO categories in males compared to females at each comparison

We identified a few thousand enhancers active in the adrenal gland at each temporal stage and in each sex (Supporting information Table S7). A subset among those belong to the super‐enhancer class (SEC), genomic regions highly enriched in active chromatin, which regulate developmental and tissue‐specific programs and often encompass disease‐associated genomic loci.^[^ ^3^ ^]^ At all ages and in both sexes, adrenal SEC have a much higher level of overlap than typical‐enhancer class (TEC) elements (Figure 2A), while adrenal TEC are significantly more conserved than SEC (Figure 2B). Adrenal SEC‐associated genes are enriched in genes involved in transcriptional regulation, cell‐cell adhesion, protein phosphorylation, biological rhythms and steroid hormone receptor function at all ages and in both sexes (Figure 2C and Supporting information Table S8). Mouse adrenal SEC‐associated genes are expressed at significantly higher levels in the adrenal gland (Supporting information Figure S4A) and include a higher percentage of tissue‐specific genes (Supporting information Figure S4B) than TEC‐associated genes. Human adrenal SEC are also preferentially associated to genes encoding proteins with adrenal‐enriched expression than to genes with higher expression in other tissues (Supporting information Figure S4C) and SEC‐associated genes are enriched with age‐dependent DEG at most times and sex‐dependent DEG at P12 weeks compared to TEC (Supporting information Figure S4D,E). Examples of SEC associated to genes important in adrenal physiology are shown in Supporting information Figure S5. Of note, some adrenal SEC‐associated genes are expressed preferentially in the adrenal and are involved in blood pressure regulation (Supporting information Table S9). In particular, non‐coding SNPs associated with blood pressure traits with very high significance are located inside a conserved SEC encompassing the *KCNK3* gene (Supporting information Figure S6 and Table S10).

**FIGURE 2:**
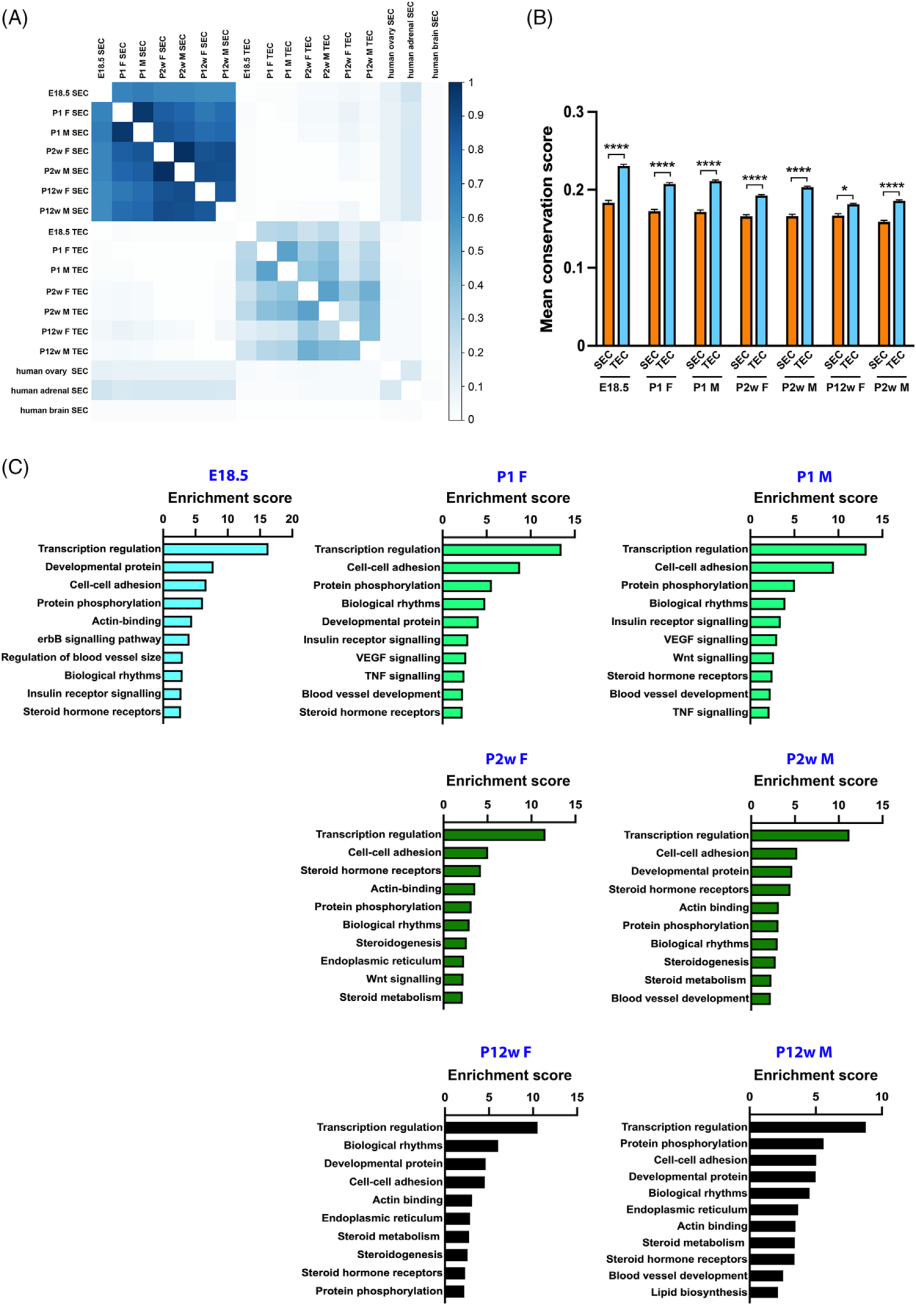
Active enhancers in the mouse adrenal gland identified by H3K27ac ChIP‐seq. (A). Pairwise comparison plot (Jaccard statistic) showing the overlap between mouse adrenal SEC, TEC and human adrenal gland, ovary and mid‐frontal lobe brain SEC. Only limited overlap exists between mouse (all ages) and human adult adrenal SEC [mean similarity coefficient (MSC) 0.17], even lower with human ovary SEC (MSC 0.09) and virtually none with human brain SEC. The effect of age on both SEC and TEC overlap is stronger (MSC 0.87 and 0.46, respectively) than the effect of sex (MSC 0.75 and 0.29, respectively). Both SEC and TEC have higher overlap at P1 (MSC 0.89 and 0.5, respectively) and P2 weeks of age (MSC 0.93 and 0.5, respectively) than at P12 weeks (MSC 0.78 and 0.39, respectively). E18.5 enhancers have more limited overlap with P2 weeks and P12 weeks enhancers than with P1 (MSC 0.60 vs. 0.73 for SEC; 0.19 vs. 0.29 or TEC). (B) Mean conservation score is higher in mouse adrenal TEC (orange) compared to SEC (sky blue) at all ages and in both sexes. *p < 0.05; ****p < 0.0001. One‐way ANOVA with Bonferroni's correction. (C) GO classification of genes associated to mouse adrenal gland SEC at each age and in both sexes

By comparing our list of adrenal sexually dimorphic DEG with data in mouse^[^ ^4^, ^5^ ^]^ and rat,^[^ ^6^ ^]^ we could highlight a core conserved sexually dimorphic gene expression program (Figure 3A and Supporting information Table S11). We focused on the role of the PRL receptor (PRLR), because of its conserved sexually dimorphic expression in the human adrenal (Figure 3B) and the well‐known role of PRL signalling in physiological adaptations and response to stress.^[^ ^7^ ^]^ All *Prlr* isoforms were upregulated in the female adrenal compared to male at P12, but not P2 weeks of age (Figure 3C). Adrenal gland weight was significantly reduced in adult female *Prlr* ‐/‐ mice compared to WT (Figure 3D), while total body weight was normal in all *Prlr* ‐/‐ animals (Supporting information Figure S7). Signalling pathway impact analysis revealed that the JAK‐STAT pathway is significantly activated in female adrenals compared to male at P12 weeks of age (Supporting information Figure S8). The mean area of adrenocortical cells in female *Prlr* ‐/‐ adrenals was significantly decreased (Figure 3E,F). This is consistent with significantly reduced circulating corticosterone levels and a trend toward an increased ACTH/corticosterone ratio in female *Prlr* ‐/‐ mice compared to WT (Figure 3G,H). These results show that PRL signalling through the PRLR has a crucial role in shaping the sexually dimorphic mouse adrenal phenotype.

**FIGURE 3:**
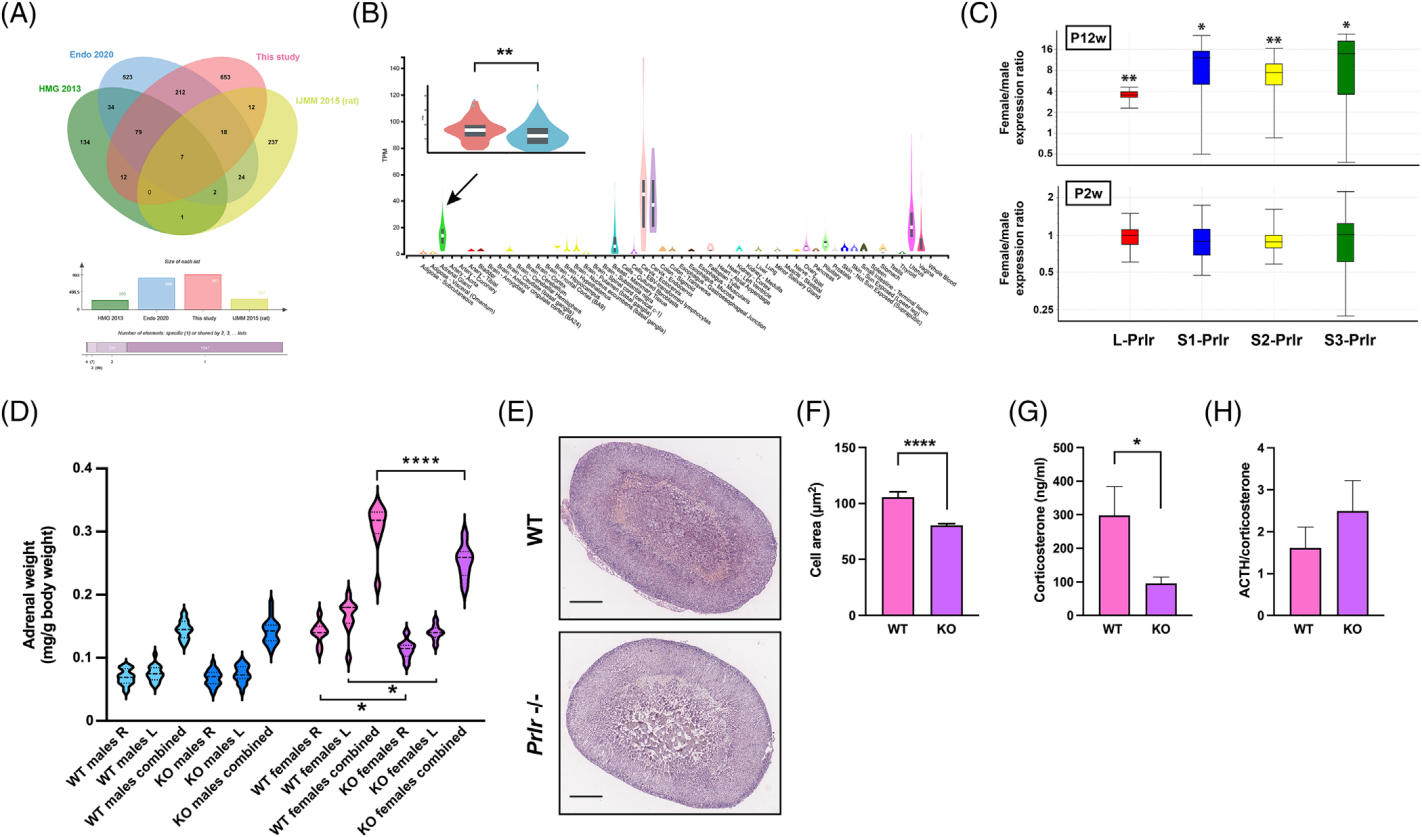
Sexually dimorphic expression of *Prlr* in the adrenal gland. (A) Overlap of sex‐dependent DEG in mouse ^(^ ^4^, ^5^ ^, and this study)^ and rat ^[^ ^6^ ^]^ adult adrenal gland evidences a core sexually dimorphic expression program. (B) Sexual dimorphic *PRLR* expression in the human adrenal gland. Data from the GTEx database. PRLR has the highest expression in the adrenal gland (indicated by an arrow) outside the female reproductive system. Inset: PRLR expression in female (red) versus male (blue) human adrenal gland. n = 71 men and n = 57 women. **p < 0.01, t‐test. (C) RT‐qPCR confirmed sexually dimorphic expression of all isoforms (long, L and short 1–3, S1‐S3) of the *Prlr* transcript in the mouse adrenal gland at P12 weeks but not P2 weeks of age. At P2 weeks, the relative percentages of the *Prlr* isoforms were: Males: L‐*Prlr* 89.7%; S1‐*Prlr* 0.1%; S2‐*Prlr* 2.2%; S3‐*Prlr* 8%. Females: L‐*Prlr* 91%; S1‐*Prlr* 0.1%; S2‐*Prlr* 2%; S3‐*Prlr* 6.9%. At P12 weeks, the relative percentages of the *Prlr* isoforms were: Males: L‐*Prlr* 94%; S1‐*Prlr* < 0.1%; S2‐*Prlr* 1%; S3‐*Prlr* 5%. Females: L‐*Prlr* 88%; S1‐*Prlr* 0.2%; S2‐*Prlr* 1.6%; S3‐*Prlr* 10.2%. *n *= 7, P2 weeks males; *n *= 5, P2 weeks females; *n *= 6, P12 weeks males; *n *= 6, P12 weeks females. *p < 0.05; **p < 0.01. Group‐wise comparison by REST. (D) Violin plot showing the body weight‐normalized adrenal gland (right, R; left, L and combined) weight for WT and *Prlr* ‐/‐ (KO) animals of both sexes at P12 weeks of age. A statistically significant difference existed in adrenal weight between WT and *Prlr* ‐/‐ females (R, L and combined). *n *= 19, WT males; *n *= 9, WT females; *n *= 18, KO males; *n *= 7, KO females. *p < 0.05; ****p < 0.0001. One‐way ANOVA with Bonferroni's correction. (E) Hematoxylin‐eosin stained adrenal gland sections from WT (top) and *Prlr* ‐/‐ (bottom) mice. Scale bar, 200 μm. (F) Mean cell area is significantly reduced in the adrenal cortex of female *Prlr* ‐/‐ mice (dark pink) compared to WT (light pink). *n *= 40 high‐power microscopic field for both WT and *Prlr* ‐/‐ females. Mean ± SEM is shown. ****p < 0.0001, *t*‐test. (G) Plasma corticosterone levels and (H) ACTH/corticosterone ratio in WT (light pink) and *Prlr* ‐/‐ (dark pink) females. *n *= 9 for both WT and *Prlr* ‐/‐ animals. Mean ± SEM is shown. *p < 0.05, *t*‐test

To assess the translational relevance of the mouse model results and to investigate the role of prolactin signalling in human physiopathology, we compared circulating adrenal steroid hormone levels in patients with PRL‐secreting (prolactinoma; PRLA) and non‐functioning pituitary adenomas (NFPA) (Supporting information Figure S9 and Table S12). Dehydroepiandrosterone sulphate (DHEAS) levels were significantly higher in patients with PRLA compared to NFPA (Figure 4A). In the PRLA group, men had higher circulating PRL levels than women (Figure 4B) and the PRL/DHEAS ratio was significantly more elevated in men than in women (Figure 4C). DHEAS levels were significantly reduced after therapy with dopamine agonists which inhibit PRL, but not ACTH, secretion (Figure 4D‐F).

**FIGURE 4:**
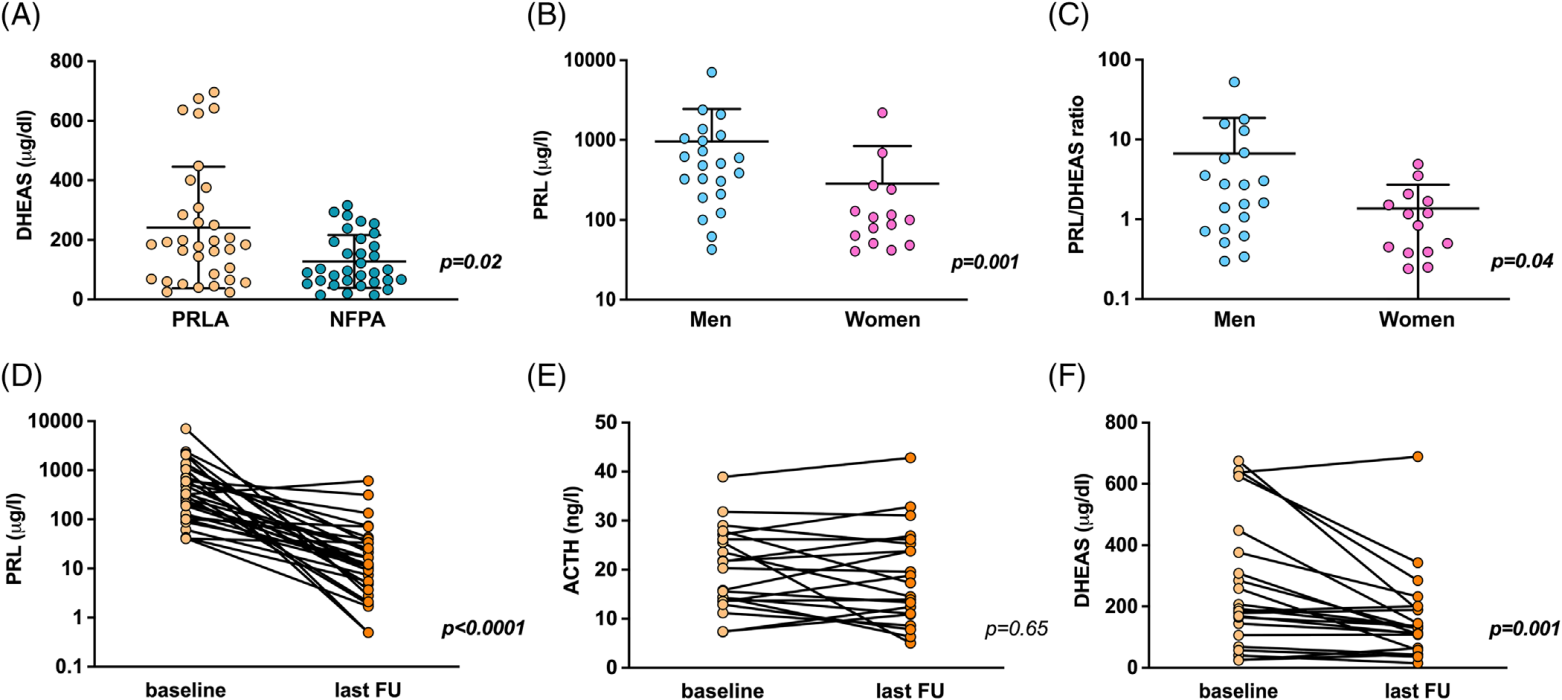
Increased circulating DHEAS levels in patients with PRLA compared to NFPA. (A) DHEAS levels were significantly elevated in patients with PRLA (*n *= 34) compared to patients with NFPA (*n *= 37). (B) PRL levels were significantly increased in male (*n *= 22) compared to female (*n *= 15) patients with PRLA. (C) The PRL/DHEAS ratio was significantly elevated in male (*n *= 20) compared to female (*n *= 14) patients with PRLA. (D) PRL levels were significantly decreased after treatment with dopamine receptor agonists in patients with PRLA (*n *= 30). Last FU = last date of follow‐up. (E) No significant variation in ACTH levels after treatment with dopamine receptor agonists in patients with PRLA (*n *= 21). Last FU = last date of follow‐up. (F) DHEAS levels were significantly decreased after treatment with dopamine receptor agonists in patients with PRLA (*n *= 22). Last FU = last date of follow‐up. Mann–Whitney test; significance values are reported in each panel

Overall, these data suggest that the female adrenal may be more sensitive to PRL effects also in humans. This parallels what we have shown in mice in this study and points on the sexual dimorphic expression of PRLR in the human adrenal gland as a key component of this increased response.^[^ ^1^, ^8^ ^]^ In humans, the effect of high PRL to preferentially increase DHEAS levels may be related to the enriched expression of PRLR in the adrenocortical *zona reticularis*.^[^ ^9^ ^]^

Modulation of adrenal steroid secretion by PRL may, thus, contribute to the positive effects of physiological concentrations of this hormone on metabolic homeostasis in basal conditions and under stress, while its deregulation in hypo‐ and hyperprolactinemic states may play an important role in the clinical manifestations of PRL deficiency or excess, respectively.

In conclusion, we have unveiled a crucial role for PRL signalling in the sexually dimorphic phenotype of the adult adrenal gland. Our results open new perspectives for the therapy of disorders characterized by adrenal hormones hypersecretion through the use of drugs modulating prolactin release.

## CONFLICT OF INTEREST

The authors declare that they have no conflict of interest.

## AUTHOR CONTRIBUTIONS

Conceptualization: E.L.; methodology: C.R, E.A., L. S.‐M., M.D.‐B., N.D., M.J., M.K.; data analysis: C.R., B.A., M.D., F.C., T.D, C.C, E.L.; supervision: T.D., C.C., E.L; writing‐original draft: E.L; writing‐review and editing: all authors.

## Supporting information



Supporting information

Supporting information

Supporting information

Supporting information

Supporting information

Supporting information

Supporting information

Supporting information

Supporting information

Supporting information

Supporting information

Supporting information

Supporting information

Supporting information

Supporting information

## Data Availability

RNA‐seq data: Gene Expression Omnibus GSE173691 (https://www.ncbi.nlm.nih.gov/geo/query/acc.cgi?acc=GSE173691). ChIP‐seq data: Gene Expression Omnibus GSE173704 (https://www.ncbi.nlm.nih.gov/geo/query/acc.cgi?acc=GSE173704).
