# Supplementary material for: Integrative genomic analysis reveals a conserved role for prolactin signalling in the regulation of adrenal function
Source: Clin Transl Med. 2021 Nov 8;11(11):e630. doi: 10.1002/ctm2.630 (PMC8574957; doi:10.1002/ctm2.630)
Supplement: Supplementary file 1 — Supporting information [file CTM2-11-e630-s005.docx]

**SUPPORTING INFORMATION**

**Supplementary Materials and Methods**

**RNA extraction from mouse adrenal glands.** All experiments were performed in accordance with the European Communities’ Council Directive 2010/63/EU. Total RNA from pooled adrenal glands of male and female C57BL6/J mice at each postnatal time [P1 males: 8 animals (16 glands); P1 females: 11 animals (22 glands); P2 week males: 3 animals (6 glands); P2 week females: 3 animals (6 glands); P12 week males: 2 animals (4 glands); P12 week females: 2 animals (4 glands)] and from timed-pregnant C57BL6/J mouse embryos at E18.5 [20 embryos (40 glands)] was isolated as described, with minor modifications^1^. Briefly, immediately after removal from the animal, adrenal glands were quickly minced on ice and transferred to Lysing Matrix D-containing tubes (MP Biomedicals) for homogenization with 500 μl of solution D (4 M guanidinium thiocyanate, 25 mM sodium citrate, pH 7, 0.5% sarkosyl, 0.1 M 2-mercaptoethanol). Samples were processed in the Fast Prep-24 Classic bead beating grinder and lysis system (MP Biomedicals) for 40 sec at a speed setting of 6.0. After centrifugation at 14,000 rpm for 5 min at 4°C, samples were transferred to new microcentrifuge tubes and incubated 5 min on ice. Sequentially, 500 μl of 2 M sodium acetate, pH 4; 500 μl of phenol (water saturated; Sigma-Aldrich), pH 4.5; and 100 μl of chloroform-isoamyl alcohol mixture (49:1; Sigma-Aldrich) were added to the homogenate with thorough mixing by inversion after the addition of each reagent. The final suspension was shaken vigorously for 10 sec and cooled on ice for 15 min. Samples were centrifuged at 14,000 rpm for 20 min at 4°C. After centrifugation, the aqueous phase was transferred to a fresh tube, mixed with 500 μl of isopropanol, and then placed at -20°C overnight to precipitate RNA. Samples were then centrifuged at 14,000 rpm for 15 min. The pellets were then washed twice with 500 μl of cold 75% ethanol, air-dried, resuspended in 30 μl of RNase-free water and incubated 10 min at 65°C. RNA concentration was determined by a NanoDrop 2000 instrument (Thermo Fisher) and its integrity was checked by running an aliquot of each sample on a denaturing 1% agarose gel stained with ethidium bromide. RNA samples to be used for RNA-seq experiments were dosed and quality assessed by Agilent 2100 Bioanalyzer (Agilent Technologies). For both reverse transcription quantitative polymerase chain reaction (RT-qPCR) and RNA-seq experiments RNA samples were subjected to DNase treatment using the DNA-free DNA Removal kit (Invitrogen) according to the manufacturer’s instructions.

**RNA-seq and data analysis.** Total RNAs were rRNA depleted using the RiboZero kit (Illumina). Libraries were prepared according to the manufacturer’s protocol (Illumina) and sequenced on a HiSeq 2500 instrument at 2x125 bp paired reads.

The FastQC tool in the Galaxy platform^2^ was used for quality control of raw fastq data. Only sequences with quality score ≥30 (Q30), as calculated by the Cutadapt tool, were further processed. Q30 reads were mapped on the mouse genome (version mm10) using HISAT2. Mapping parameters were strand: RF; - paired-end alignment: --fr; minimum intron length: 20; maximum intron length: 500.000; GTF file with known splice sites: mm10; list of novel splice sites: true. The number of reads mapping to NCBI RefSeq features were calculated by the ht-seq count tool with parameters stranded: reverse; minimum alignment quality: 10; feature type: exon. Counts were then normalized by the DESeq2 tool in Galaxy. Read statistics are reported in Table S13. The PCA plot and dendrogram of sample distribution were obtained by TCC-GUI^3^ using default parameters. DEG according to age (in pairwise sex-matched samples: E18.5 *vs.* P1; P1 *vs*. P2 weeks; P2 weeks *vs*. P12 weeks) and to sex (in pairwise age-matched samples: P1 females *vs.* males; P2 weeks females *vs.* males: P12 weeks females *vs.* males) were identified by NOISEq^4^, using the following parameters: normalization method=TMM; number of simulated replicates=5; size of the simulated replicates=0.2; variability=0.02; probability threshold=0.9 in the OmicsBox suite (BioBam). Venn diagrams were drawn using jvenn^5^ and heatmaps were produced using the heatmap2 tool in Galaxy. GO classification of DEG was performed by DAVID^6^. The percentages of immune cells infiltrating the adrenal tissue were calculated from RNA-seq data using seq-ImmuCC^7^ and the percentages of transcripts belonging to the various RNA classes were calculated by the gffcompare tool in Galaxy. RNA-seq reads were visualized with the Integrative Genomics Viewer (IGV) software (https://igv.org/).

**RT-qPCR for *Prlr* isoform expression.** A total of 500 ng of total RNA was reverse transcribed using Superscript IV Reverse Transcriptase (Invitrogen). RT-qPCR was performed using the SYBR Green I dye assay on a LightCycler 480 (Roche Applied Science) instrument using mouse TATA-binding protein (*Tbp*) as a reference transcript. Primer sequences used^8^ were as follows: *Prlr* extracellular domain (ECD) forward 5’-ATAAAAGGATTTGATACTCATCTGCTAGAG-3’; *Prlr* intracellular domain (ICD) long, reverse 5’-TGTCATCCACTTCCAAGAACTCC-3’; *Prlr* ICD S1, reverse 5’-CATAAAAACTCAGTTGTTGGAATCTTCA-3’; *Prlr* ICD S2, reverse 5’-GGAAAAAGACATGGCAGAAACC-3’; *Prlr* ICD S3 reverse, 5-’AGTTCCCCTTCATTGTCCAGTTT-3’; *Tbp* 5’-AGGCCAGACCCCACAACTC-3’ (forward) and 5’-GGGTGGTGCCTGGCAA-3’ (reverse). The relative proportions of the different *Prlr* isoforms were calculated with the 2^-ΔΔCt^ method^9^, according to the equation *x* + [1/2^-ΔΔCt^(S1-*Prlr* – L-*Prlr*) + 1/2^-ΔΔCt^(S2-*Prlr* – L-*Prlr*) + 1/2^-ΔΔCt^(S3-*Prlr* – L-*Prlr*)] = 1, where *x* is the proportion of the L-*Prlr* transcript. *PRLR* expression data in the human adrenal gland were retrieved from the GTEx database (<https://www.gtexportal.org/home/gene/PRLR>).

***Prlr* null mouse experiments.** C57BL/6 mice heterozygous for the PRL receptor (*Prlr* +/-) from The Jackson Laboratory^10^ were colony expanded and maintained in the animal facility of the Institute of Neurobiology of the National University of Mexico (UNAM). The Bioethics Committee of the Institute of Neurobiology (UNAM) approved all animal experiments. Analyses were carried out in young adult (12 weeks of age) female and male mice null (*Prlr* -/-) or WT (*Prlr* +/+) for the PRL receptor. Animals were fed *ad libitum* and reared in standard laboratory conditions (22°C, 12 h/12 h light/dark cycle). Mice were euthanized by CO_2_ inhalation and decapitation between 11 am and 1 pm. Adrenal glands were dissected from the surrounding fat tissue and weighed. Morphological analysis of adrenal glands from *Prlr* +/+ and -/- females was performed by a custom Fiji (https://imagej.net/Fiji) plugin developed to count the number of cell nuclei in the adrenal cortex, as detected by hematoxylin staining of tissue sections, per area unit. 10 randomly chosen high-power microscopic fields were analyzed for 4 animals per genotype. Corticosterone and ACTH were assayed using commercial ELISA kits (Abcam).

**Hormone measurements in patients with PRLA and NFPA.** Patients with PRLA (*n*=37; 22 men and 15 women) and NFPA (*n*=37; 22 men and 15 women) were evaluated for plasma steroid hormone levels. Inclusion criteria were: diagnosis of PRLA and hyperprolactinemia at the time of hormone measurement for the PRLA group; diagnosis of NFPA and normal circulation levels of PRLA for the NFPA group. Exclusion criteria for both groups were: concomitant therapy with glucocorticoids (including hydrocortisone substitution) and/or anti-androgens, ACTH levels above normal values (to exclude PRL-ACTH co-secretion by the tumor), hypopituitarism secondary to pituitary lesions leading to adrenal insufficiency and plasma samples older than 9 years before measurement. Clinical data, including symptoms and medical treatment as well as hormonal levels were collected for all patients. Patients with PRLA and NFPA were matched for sex, age, BMI and tumor size. The protocol was approved by the Ethics Committee of the University of Würzburg (#85/12) and informed consent was obtained from the patients involved in the study. PRL, ACTH, cortisol and DHEAS were measured by the automated immunoanalysis Immulite XPi2000 system (Siemens). Aldosterone, cortisol, cortisone, corticosterone, 11-deoxycortisol, testosterone, androstenedione, DHEA, and 17-OH progesterone (due to concentrations smaller than the lower limit of quantitation in most patients, 21-deoxycortisol, estradiol, dihydrotestosterone, 11-deoxycorticosterone and progesterone were excluded from further analysis) were measured by liquid chromatography-tandem mass spectrometry (LC-MS/MS) at the basal state and 1 hour after injection of 250 μg ACTH. In addition, luteinizing hormone (LH), follicle-stimulating hormone (FSH), thyroid stimulating hormone (TSH), free thyroxine (FT4) and insulin-like growth factor-1 (IGF1) were measured by Immulite in both groups. To note, for the final analysis, TSH, FT4 and testosterone were evaluated only in those patients who were not treated with specific hormone supplementation, whereas aldosterone levels were evaluated only in patients who were not treated with drugs known to interfere with the renin-angiotensin-aldosterone system (including sartans, angiotensin converting-enzyme-inhibitors, diuretics and beta-blockers). For the PRLA group, PRL levels after dopamine agonist treatment were collected in 30 patients at the last follow-up [median time from the baseline was 47 (range 2-118) months]. Among this subgroup, 22 patients also had data available on ACTH and DHEAS levels.

**Tissue dissection and chromatin immunoprecipitation (ChIP).** ChIP was performed as previously described^11,12^ and optimized for mouse tissues^13^. Adrenal glands from male and female C57BL6/J mice were dissected on ice at each postnatal developmental stage [P1 males and females: 11 animals (22 glands) each; P2 week males and females: 6 animals (12 glands) each; P12 week males and females: 3 animals (6 glands) each]. To determine the sex of P1 mice, PCR amplification from mouse tails was used for the detection of the male-specific sex determining region Y (*Sry*) gene employing the following specific primers: forward 5’-TGGGACTGGTGACAATTGTC-3’ and reverse 5’-GAGTACAGGTGTGCAGCTCT-3’. Embryonic adrenal glands were isolated from timed-pregnant C57BL6/J strain mouse embryos at E18.5 by microdissection in cold phosphate buffered saline (PBS) and pooled (for a total of 30 glands) without determining the sex of the embryos. Dissected tissues were then transferred to 1.5 ml microcentrifuge tubes containing PBS plus Complete Protease Inhibitor Cocktail (PIC, Roche) and crosslinked with 1% formaldehyde added directly to the PBS for 15 min at room temperature on a rotating wheel. After quenching with 0.25 M glycine, tissue samples were washed twice with PBS, resuspended in Lysis Buffer I [50 mM HEPES-KOH (pH 7.5), 140 mM NaCl, 1 mM EDTA,10% glycerol, 0.5% NP-40, 0.25% Triton X-100, containing PIC and 1 mM phenylmethanesulfonyl fluoride (PMSF)] and transferred in a glass douncer for tissue lysis on ice. After homogenization, the lysates were transferred to the original tubes, put on a rotating wheel for 10 min and centrifuged at 1,400*g* for 5 min at 4°C. Pellets were resuspended in Lysis Buffer II [10 mM Tris-HCl (pH 8.0), 200 mM NaCl, 1 mM EDTA, 0.5 mM EGTA, supplemented with PIC and 1 mM PMSF], put on a rotating wheel and centrifuged as previously described, lysed with Lysis Buffer III [10 mM Tris-HCl (pH 8.0), 100 mM NaCl, 1 mM EDTA, 0.5 mM EGTA, 0.1% Na-Deoxycholate, 0.5% N-lauroylsarcosine, containing PIC and 1 mM PMSF] and sonicated for a minimum of 20 to a maximum of 30 cycles at 20% amplitude (30 sec sonication, 60 sec rest) with a Branson digital sonifier to shear chromatin to a final average size of ~200 bp. After centrifugation to pellet debris, ChIP was performed incubating the supernatant overnight at 4°C on a rotating wheel with 10 μg of anti-lysine 27 acetylated histone H3 antibody (H3K27ac, Abcam #4729), previously coupled to immunomagnetic protein A beads (Dynabeads, Invitrogen). The magnetic beads carrying the immunoprecipitated chromatin were washed five times with RIPA buffer [50 mM HEPES–KOH (pH 7.5), 500 mM LiCl, 1 mM EDTA, 1% NP-40, 0.7% Na-deoxycholate supplemented with PIC and 1 mM PMSF] and once with TE buffer [10 mM Tris-HCl, pH 8, 1 mM EDTA] containing 50 mM NaCl. DNA was eluted by incubation with elution buffer [50 mM Tris–HCl (pH 8.0), 10 mM EDTA, 1% SDS] at 65°C for 25 min and incubated at 65°C overnight to reverse cross-links. We systematically checked for successful immunoprecipitation of histone H3 acetylated on Lys27. After the reversal of cross-linking, immunoprecipitated DNA was purified by RNAse A (Sigma, 0.2 mg/ml final concentration) treatment and proteinase K (Invitrogen, 0.2 U/ml final concentration) digestion, phenol-chloroform extracted, precipitated, resuspended in 10 mM Tris–HCl (pH 8.0) and quantified on a Qubit fluorometer (Invitrogen). For ChIP of embryonic adrenal glands we used the ChIP-IT High Sensitivity kit (Active Motif) according to the manufacturer's instruction. Briefly, tissues were fixed with a specially formulated formaldehyde buffer on a rotating wheel for 15 min at room temperature. The fixation reaction was stopped by the addition of a stop solution on a rotating wheel for 5 min at room temperature. Tissues were then centrifuged for 3 min at 1,250*g* at 4°C and pellets were washed twice with ice-cold PBS Wash Buffer, resuspended in Chromatin Prep Buffer supplemented with PIC and 1 mM PMSF, incubated on ice for 10 min, then transferred to a glass douncer for homogenization. After centrifugation for 3 min at 1,250*g* at 4°C, pellets were resuspended in a ChIP Buffer containing PIC and 1 mM PMSF, incubated on ice for 10 min and subjected to sonication as described above. After centrifugation to pellet debris, ChIP was performed incubating the sonicated chromatin overnight at 4°C on a rotating wheel with 4 μg of anti-H3K27ac antibody, as described before. Antibody-bound protein/DNA complexes were immunoprecipitated by incubating ChIP reactions with Protein G agarose beads overnight at 4°C on a rotating wheel, then transferred to ChIP Filtration Columns for washing and elution. Eluted ChIP DNA was subjected to cross-link reversal and proteinase K digestion, recovered and purified on specific DNA purification columns. Quantification was performed by Qubit fluorimetry as indicated above.

**High-throughput sequencing of immunoprecipited DNA and data analysis.** Libraries were prepared from ChIPped DNA according to the manufacturer’s protocol (Illumina TruSeq ChIP kit) and sequenced on a HiSeq 2500 instrument at 1x50 bp single-end reads. The FastQC tool in the Galaxy platform^2^ was used for quality control of raw fastq data. Only sequences with quality score ≥30 (Q30), as calculated by the Cutadapt tool, were further processed. Q30 reads

were mapped on the mouse genome (version mm10) using Bowtie2 in Galaxy. Peaks were called by MACS2 in the Galaxy platform using genomic mouse DNA as control by its broad peak algorithm and minimum FDR (q-value) cutoff for peak detection = 0.05. Read statistics are reported in Table S14. H3K27ac-enriched regions were stitched together at a maximum distance of 12.5 kb, with exclusion of the peaks lying within 1 kb from gene TSS. Enhancers were classified as typical enhancer class (TEC) and super-enhancer class (SEC) on the basis of the intensity of their ChIP signals using the ROSE software^14^. This algorithm ranks the enhancers by their input-subtracted H3K27ac signal, then separates SEC from TEC by identifying an inflection point of the H3K27ac signal graph plotted against the enhancer rank. Enhancers were annotated to the next nearby genes using PAVIS^15^. Coordinates of the human adrenal gland, ovary and brain mid frontal lobe SEC were downloaded from the dbSUPER database^16^ and mapped in mm10 using the LiftOver tool in the UCSC Genome Browser (<http://genome.ucsc.edu/cgi-bin/hgLiftOver>). The overlap between SEC and TEC enhancers was calculated and plotted using the pairwise intersection tool^17^ in Galaxy. Enhancer conservation was calculated according to phastCons60way scores for mm10. GO classification of enhancer-associated genes was performed by DAVID^6^. The lists of adrenal-enriched genes were retrieved from ref. 18 and ref. 19 for mouse and human, respectively. For the analysis of the association of adrenal gland SEC to GWAS SNPs related to BP traits, the list of genes associated to BP SNPs^20^ was intersected with the lists of human adrenal SEC-associated genes and of the adrenal-enriched genes^19^.

**Statistical analysis.** To assess the similarity between samples, a Bray-Curtis similarity matrix was generated using the PRIMER 7 software (PRIMER-e). A permutational multivariate analysis of variance (PERMANOVA)^21^ was used to determine the statistical significance of differences in global transcriptional profiles. A sample resemblance matrix was derived after fourth root transformation following the Bray-Curtis algorithm and using type III (partial) sums of squares with a fixed effects sum to zero for mixed terms. Exact P values were generated using unrestricted permutation of raw data (9999 permutations). Pseudo-F statistic and generated P values were reported for each condition where differences in gene expression were observed. Further pairwise tests were also conducted using PERMANOVA. Signalling pathway impact analysis (SPIA) was performed as described^22^ using the Graphite Web webtool^23^. Fisher's exact test, χ^2^ with Yates’ correction, *t*-test, Mann-Whitney test and one-way ANOVA were performed as indicated in the figure legends using Prism 9.1 (GraphPad). The Relative Expression Software Tool (REST; Qiagen) was used for analysis of relative expression results in RT-qPCR experiments. The χ^2^ test was performed for dichotomic variables of patients’ data using the SPSS Software (PASW Version 21.0, SPSS Inc.). A level of *p*<0.05 was considered as statistically significant.

**References**

1. Chomczynski P, Sacchi N. Single-step method of RNA isolation by acid guanidinium thiocyanate-phenol-chloroform extraction. *Anal Biochem*. 1987;162(1):156-159.

2. Jalili V, Afgan E, Gu Q, Clements D, Blankenberg D, Goecks J, Taylor J, Nekrutenko A. The Galaxy platform for accessible, reproducible and collaborative biomedical analyses: 2020 update. *Nucleic Acids Res*. 2020;48(14):W395-W402.

3. Su W, Sun J, Shimizu K, Kadota K. TCC-GUI: a Shiny-based application for differential expression analysis of RNA-Seq count data. *BMC Res Notes.* 2019;12(1):133.

4. Tarazona S, García-Alcalde F, Dopazo J, Ferrer A, Conesa A. Differential expression in RNA-seq: a matter of depth. *Genome Res*. 2011;21(12):2213-2223.

5. Bardou P, Mariette J, Escudié F, Djemiel C, Klopp C. jvenn: an interactive Venn diagram viewer. *BMC* *Bioinformatics*. 2014;15(1):293.

6. Huang DW, Sherman BT, Lempicki RA. Systematic and integrative analysis of large gene lists using DAVID Bioinformatics Resources. *Nat Protoc.* 2009;4(1):44-57.

7. Chen Z, Quan L, Huang A, et al. seq-ImmuCC: cell-centric view of tissue transcriptome measuring cellular compositions of immune microenvironment from mouse RNA-seq data. *Front Immunol.* 2018;9:1286.

8. Ferraris J, Boutillon F, Bernadet M, Seilicovich A, Goffin V, Pisera D. Prolactin receptor antagonism in mouse anterior pituitary: effects on cell turnover and prolactin receptor expression. *Am J Physiol Endocrinol Metab.* 2012;302(3):E356-E364.

9. Livak KJ, Schmittgen TD. Analysis of relative gene expression data using real-time quantitative PCR and the 2^-ΔΔC^_T_ method. *Methods*. 2001;25(4):402-408.

10. Ormandy CJ, Camus A, Barra J et al. Null mutation of the prolactin receptor gene produces multiple reproductive defects in the mouse. *Genes Dev*. 1997;11(2):167-178.

11. Lee TI, Johnstone SE, Young RA. Chromatin immunoprecipitation and microarray-based analysis of protein location. *Nat Protoc*. 2006;1(2):729-748.

12. Doghman M, Figueiredo BC, Volante M, Papotti M, Lalli E. Integrative analysis of SF-1 transcription factor dosage impact on genome-wide binding and gene expression regulation. *Nucleic Acids Res*. 2013;41(19):8896-8907.

13. Visel A, Blow MJ, Li Z, et al*.* ChIP-seq accurately predicts tissue-specific activity of enhancers. *Nature.* 2009*;*457(7231):854-858.

14. Hnisz D, Abraham BJ, Lee TI, Lau A, Saint-André V, Sigova AA, Hoke HA, Young RA. Super-enhancers in the control of cell identity and disease. *Cell.* 2013*;*155(4):934-947.

15. Huang W, Loganantharaj R, Schroeder B, Fargo D, Li L. PAVIS: a tool for Peak Annotation and Visualization. *Bioinformatics.* 2013;29(23):3097-3099.

16. Khan A, Zhang X. dbSUPER: a database of super-enhancers in mouse and human genome. *Nucleic Acids Res*. 2016;44(D1):D164-D171.

17. Khan A, Matelier A. Intervene: a tool for intersection and visualization of multiple gene or genomic region sets. *BMC Bioinformatics.* 2017;18(1):287.

18. Li B, Qing T, Zhu J, Wen Z, Yu Y, Fukumura R, Zheng Y, Gondo Y, Shi L. A comprehensive mouse transcriptomic BodyMap across 17 tissues by RNA-seq. *Sci Rep*. 2017;7(1):4200.

19. The Human Protein Atlas 2021; https://www.proteinatlas.org/humanproteome/tissue/ adrenal+gland.

20. GWAS Catalog 2021; https://www.ebi.ac.uk/gwas/efotraits/EFO_0004325.

21. Anderson MJ. A new method for non-parametric multivariate analysis of variance. *Austral Ecol*. 2001; 26(1):32– 46.

22. Tarca AL, Draghici S, Khatri P, Hassan SS, Mittal P, Kim JS, Kim CJ, Kusanovic JP, Romero R. A novel signaling pathway impact analysis. *Bioinformatics*. 2009;25(1):75-82.

23. Sales G, Calura E, Martini P, Romualdi C. Graphite Web: web tool for gene set analysis exploiting pathway topology. *Nucleic Acids Res*. 2013; 41(Web Server issue):W89-97.

24. Hanemaaijer ES, Margaritis T, Sanders K, et al. Single-cell atlas of developing murine adrenal gland reveals relation of Schwann cell precursor signature to neuroblastoma phenotype. *Proc Natl Acad Sci U S A.* 2021*;*118(5):e2022350118.

25. Lai S, Ma L, E W, Ye F, Chen H, Han X, Guo G. Mapping a mammalian adult adrenal gland hierarchy across species by microwell-seq. *Cell Regen.* 2020*;*9(1):11.

26. Lopez JP, Brivio E, Santambrogio A, et al. Single-cell molecular profiling of all three components of the HPA axis reveals adrenal ABCB1 as a regulator of stress adaptation. *Sci Adv*. 2021;7(5):eabe4497.

**Supplementary Figures**

**
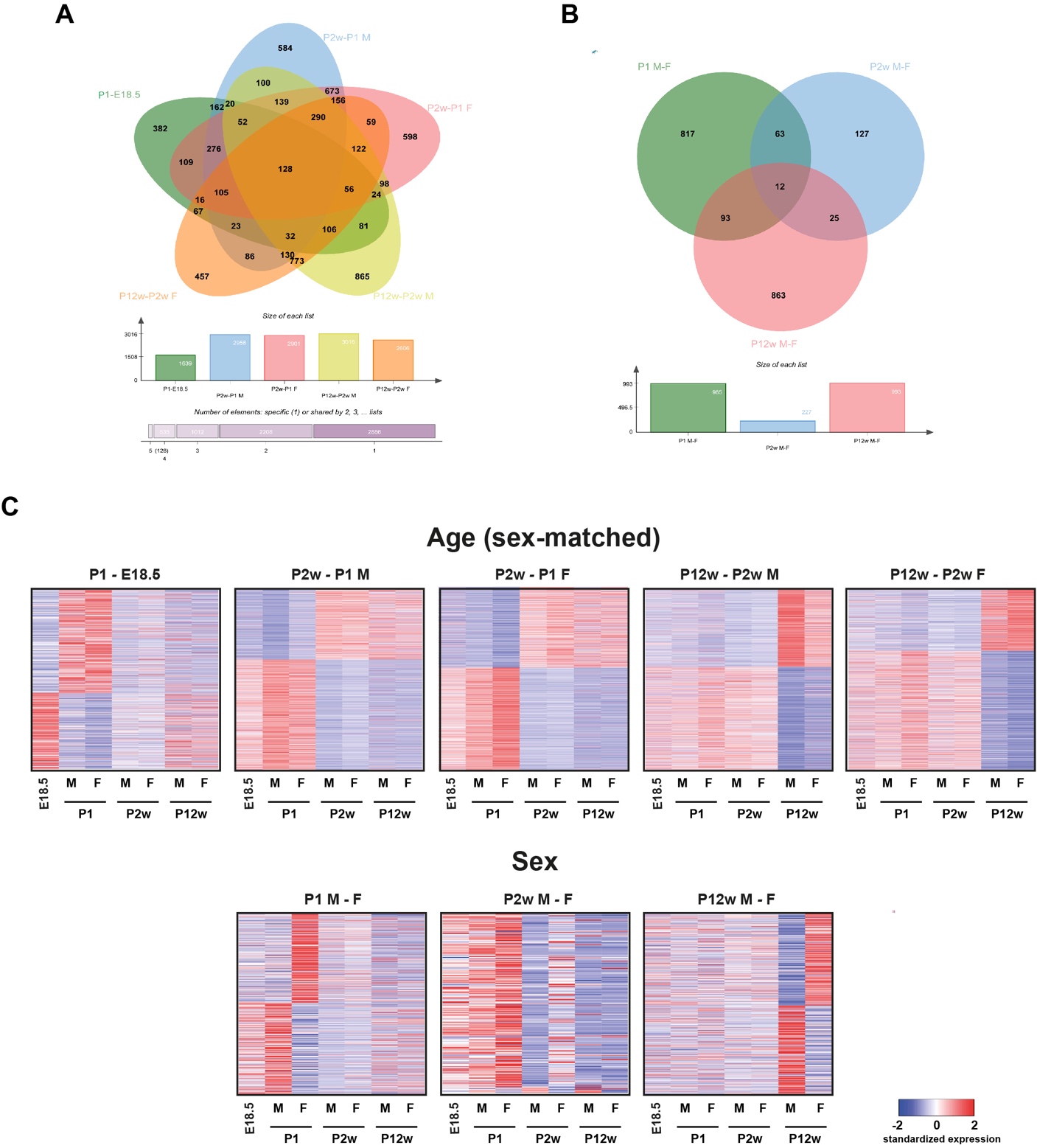
**

Figure S1. Age- and sex-dependent gene expression profiles in the mouse adrenal gland. (A) Venn diagram of DEG according to animal age in sex-matched animals. The number of elements in each list and the number of common elements in different numbers of lists are shown with histograms. **(**B) Venn diagram of DEG according to animal sex. The number of elements in each list is shown with histograms. **(**C) Heatmaps of age-dependent and sex-dependent DEG expression in the different samples.

**
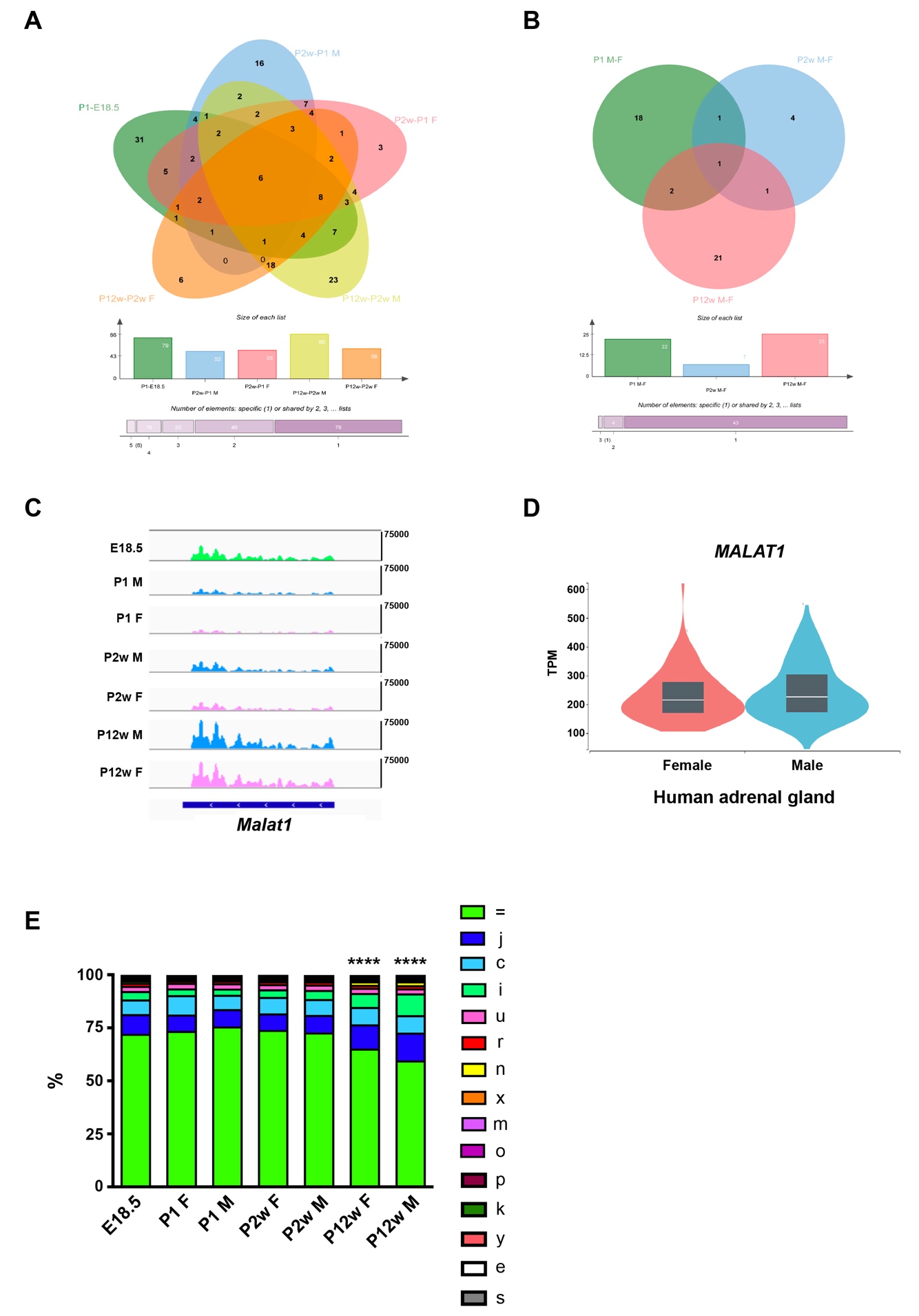
**

Figure S2. Differential expression of ncRNAs in the mouse adrenal gland. (A) Venn diagram of differentially expressed ncRNAs among sex-matched samples according to animal age. (B) Venn diagram of differentially expressed ncRNAs according to animal sex. (C) IGV snapshot of RNA-seq reads in the mouse -adrenal gland at different ages and in both sexes across the non-coding *Malat1* gene. (D) *MALAT1* expression in the human adrenal gland (data from the GTEx portal). (E) Transcripts classification (all classes) in the mouse adrenal glands across the various ages and in both sexes. Transcripts are classified according to the gffcompare nomenclature: *=*, complete, exact match of intron chain; *j*, multi-exon with at least one junction match (potentially novel isoform); *c*, predicted transcript is contained in the reference transcript; *i,* predicted transcript falls entirely within a reference intron; *u*, predicted transcript is intergenic in comparison with known reference transcripts; *r*, predicted transcript has >50% of its bases overlapping a soft-masked (repetitive) reference sequence; *n*, transcript has retained introns or not all introns are matched/covered; *x*, exon of predicted transcript overlaps reference but lies on the opposite strand; *m*, retained intron(s), all introns matched or retained; *o,* exon of predicted transcript overlaps a reference transcript; *p*, predicted transcript lies within 2 kb of a reference transcript (possible polymerase run-on transcript); *k*, containment of reference; *y*, transcript contains a reference transcript within its introns; *e*, predicted single-exon transcript overlaps a reference exon plus at least 10 bp of a reference intron, indicating a possible pre-mRNA fragment; *s*, intron of predicted transcript overlaps a reference intron on the opposite strand. **** *p*<0.0001 for the abundance of = class transcripts compared to the other categories. χ^2^ with Yates’ correction.

**
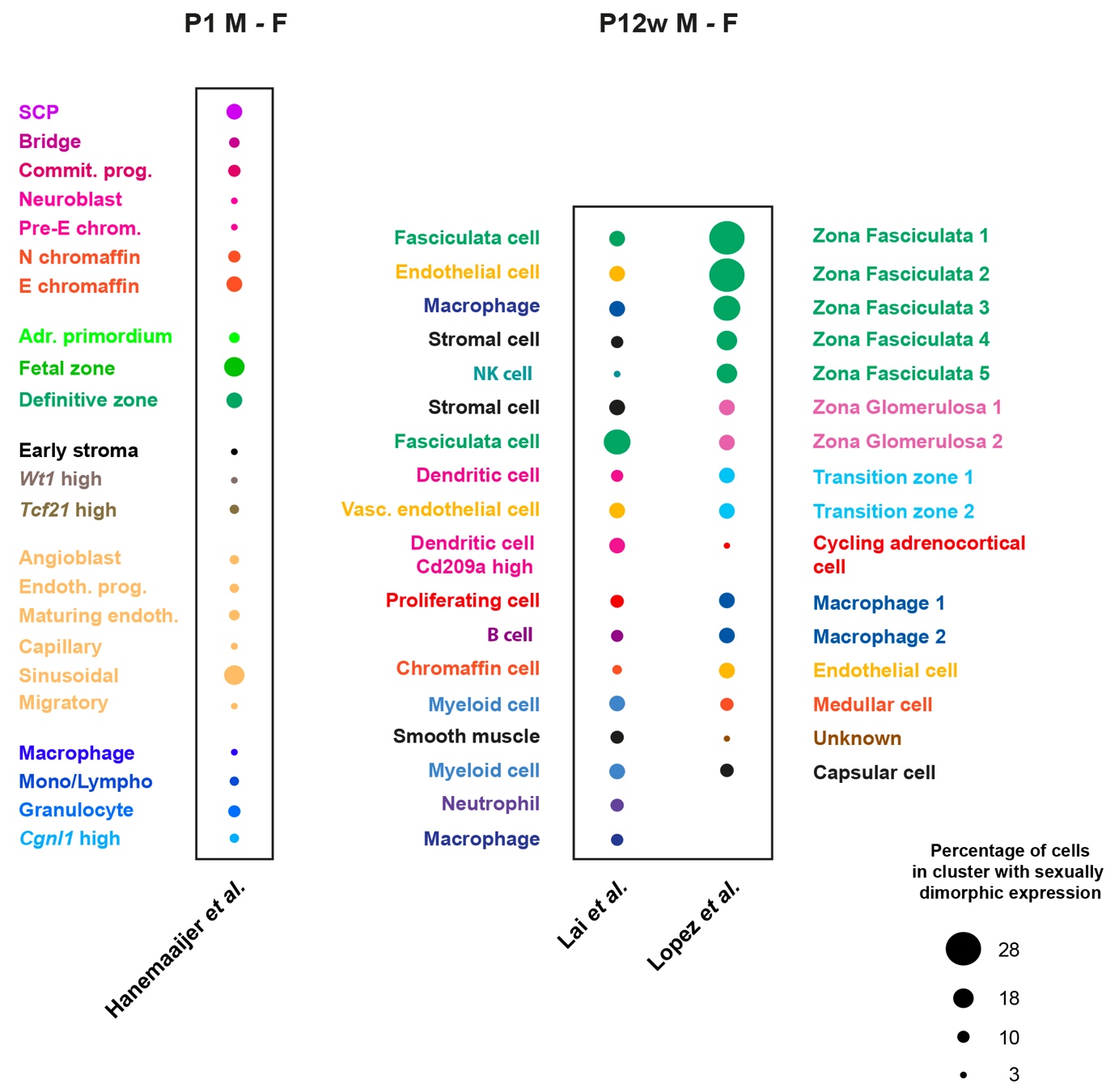
**

Figure S3. Overlap of sex-dependent DEG with cell populations identified by previous studies using scRNA-seq in the early postnatal and adult mouse adrenal gland. Data from Hanemaajier et al.^24^, Lai et al.^25^ and Lopez et al.^26^

**
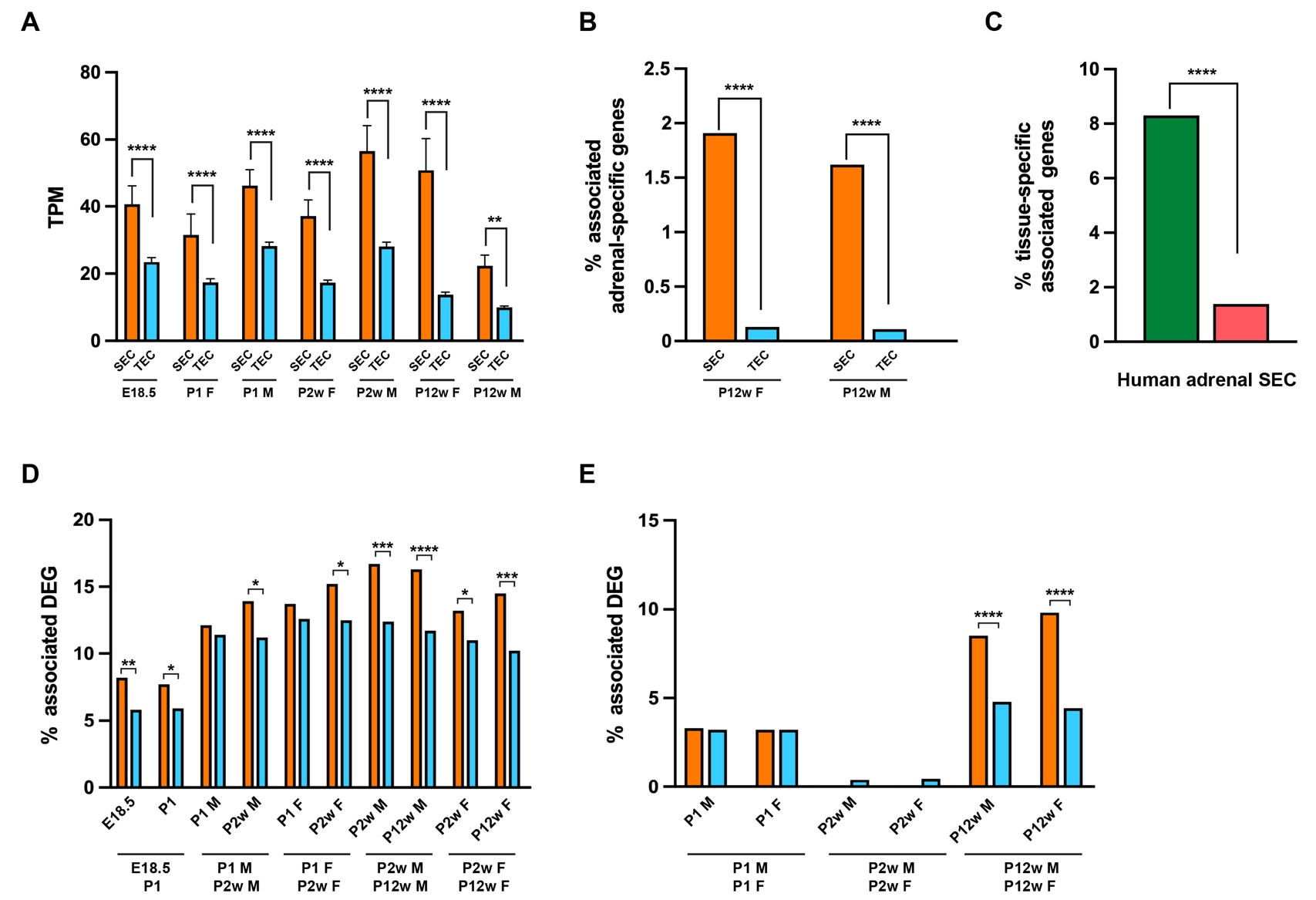
**

Figure S4. SEC are preferentially associated to highly expressed, tissue-specific and differentially expressed genes in the adrenal gland. (A) Mouse adrenal SEC (orange) are associated to more highly expressed genes compared to TEC (sky blue). Mean ±SEM is shown. ***p*<0.01; *****p*<0.0001. One-way ANOVA with Bonferroni’s correction. (B) Mouse adrenal SEC (orange) are preferentially associated to adrenal-enriched genes compared to TEC (sky blue) *****p*<0.0001. χ^2^ with Yates’ correction. (C) Adrenal gland SEC are preferentially associated to adrenal-enriched genes (green) than to genes whose expression is enriched in other tissues (red). *****p*<0.0001. χ^2^ with Yates’ correction. (D) Mouse adrenal SEC (orange) are preferentially associated to age-dependent DEG compared to TEC (sky blue). **p*<0.05; ***p*<0.01; ****p*<0.001; *****p*<0.0001. Fisher’s exact test and χ^2^ with Yates’ correction. (E) Mouse adrenal SEC (orange) are preferentially associated to sex-dependent DEG compared to TEC (sky blue) at P12 weeks but not at earlier ages. *****p*<0.0001. χ^2^ with Yates’ correction.

**
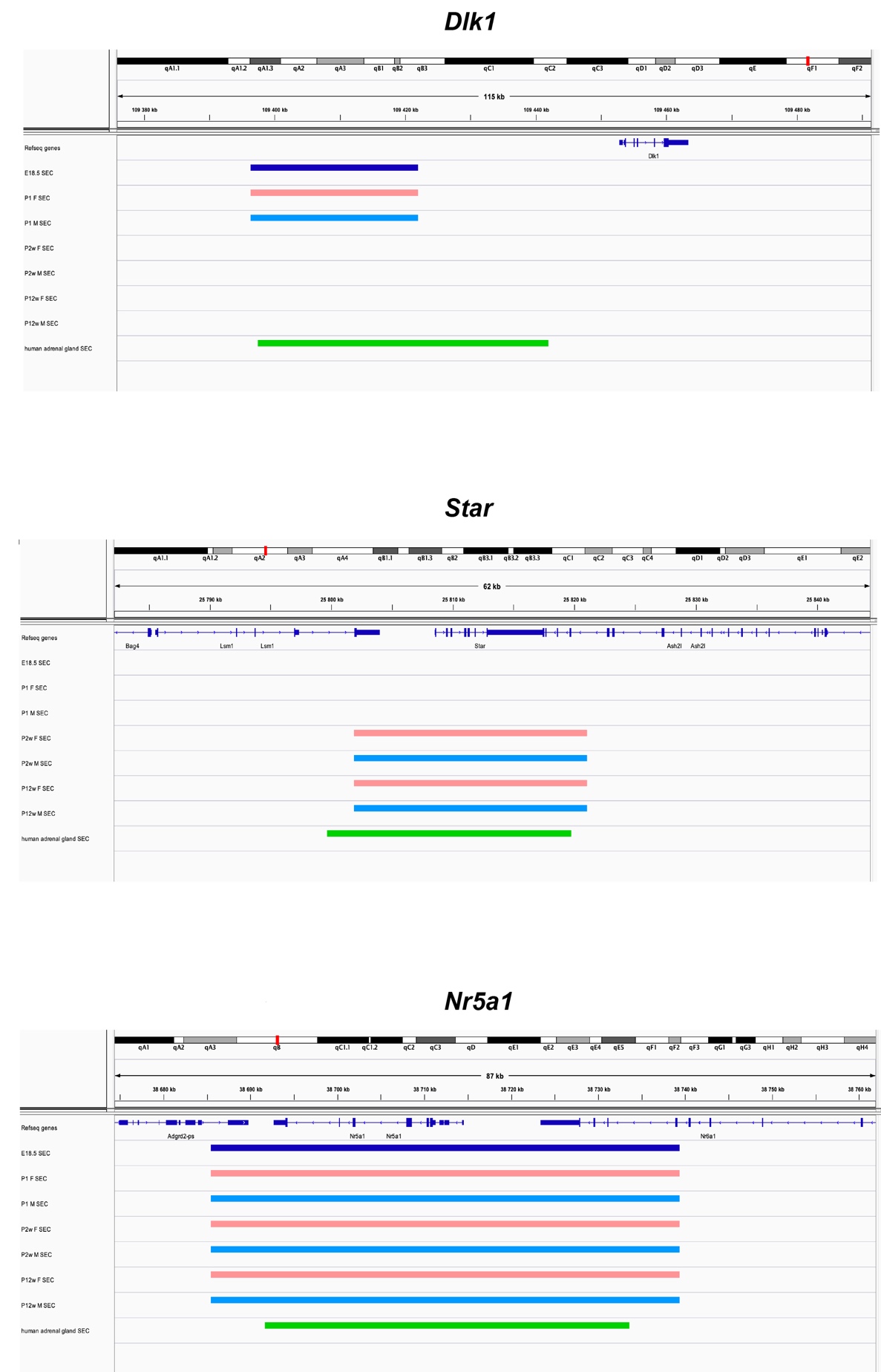
**

Figure S5. IGV snaphots of mouse and human adrenal SEC in the genomic regions of the *Dlk1* (top), *Star* (middle) and *Nr5a1* (bottom) genes.

**
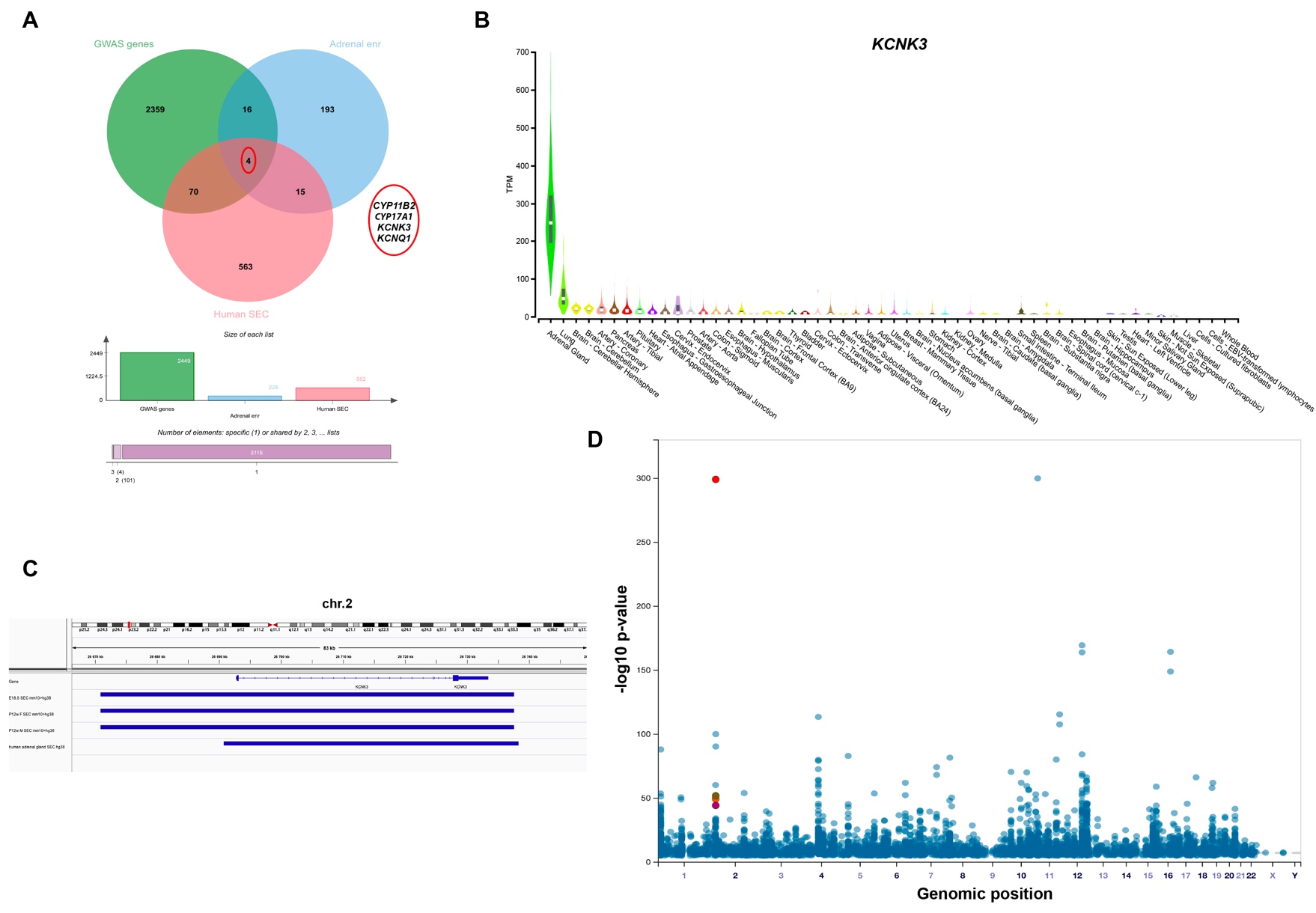
**

Figure S6. Human adrenal SEC association with loci implicated in blood pressure (BP) traits. (A) Venn diagram showing intersection of the lists of: genes associated to SNPs identified in BP GWAS studies; adrenal-enriched genes; human adrenal SEC-associated genes. The number of elements in each list and the number of common elements in different numbers of lists are shown with histograms. Only 4 genes (*CYP11B2*, *CYP17A1*, *KCNK3* and *KCNQ1*) are in common among the three lists. (B) *KCNK3* has the highest expression in the adrenal gland among all human tissues. Data from the GTEx portal. (C) IGV snapshot of the position of the conserved SEC in mouse (E18.5 and adult, males and females) and human which extends upstream of the *KCNK3* gene, where the four -associated SNPs are localized. (D) Plot showing the genomic localization of SNPs associated to BP traits. In chromosome 2, the positions of the *KCNK3* upstream SNPs rs1275984 (red), rs1275988 (brown), rs1275985 (orange) and rs1275986 (purple) are highlighted. From ref. 20.


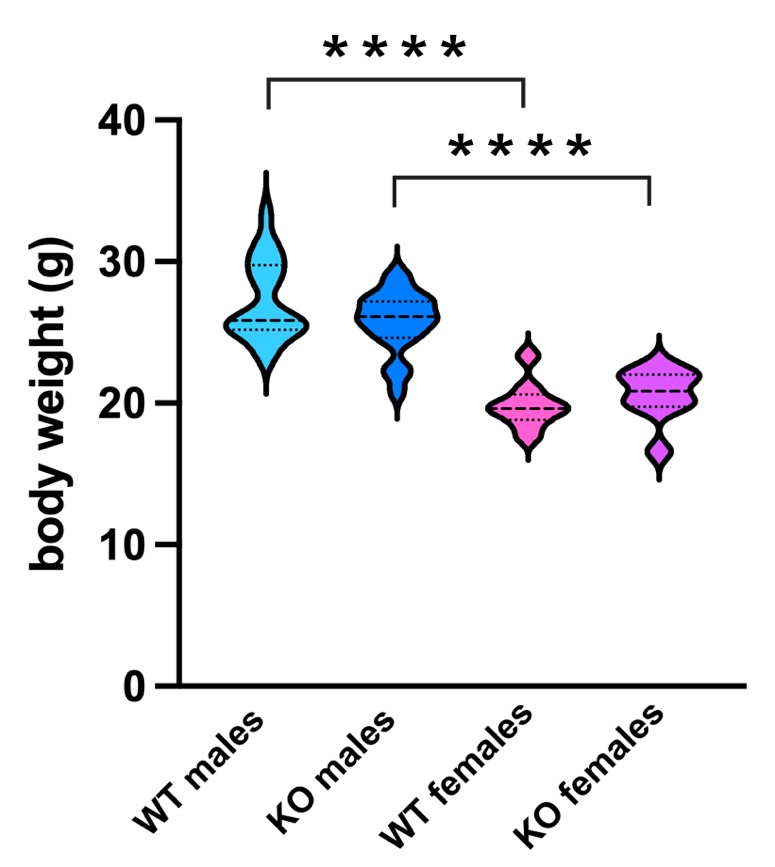


Figure S7. Body weight of WT and *Prlr* -/- (KO) mice. *n*=19, WT males; *n*=9, WT females; *n*=18, KO males; *n*=7, KO females. *****p*<0.0001. One-way ANOVA with Bonferroni’s correction.


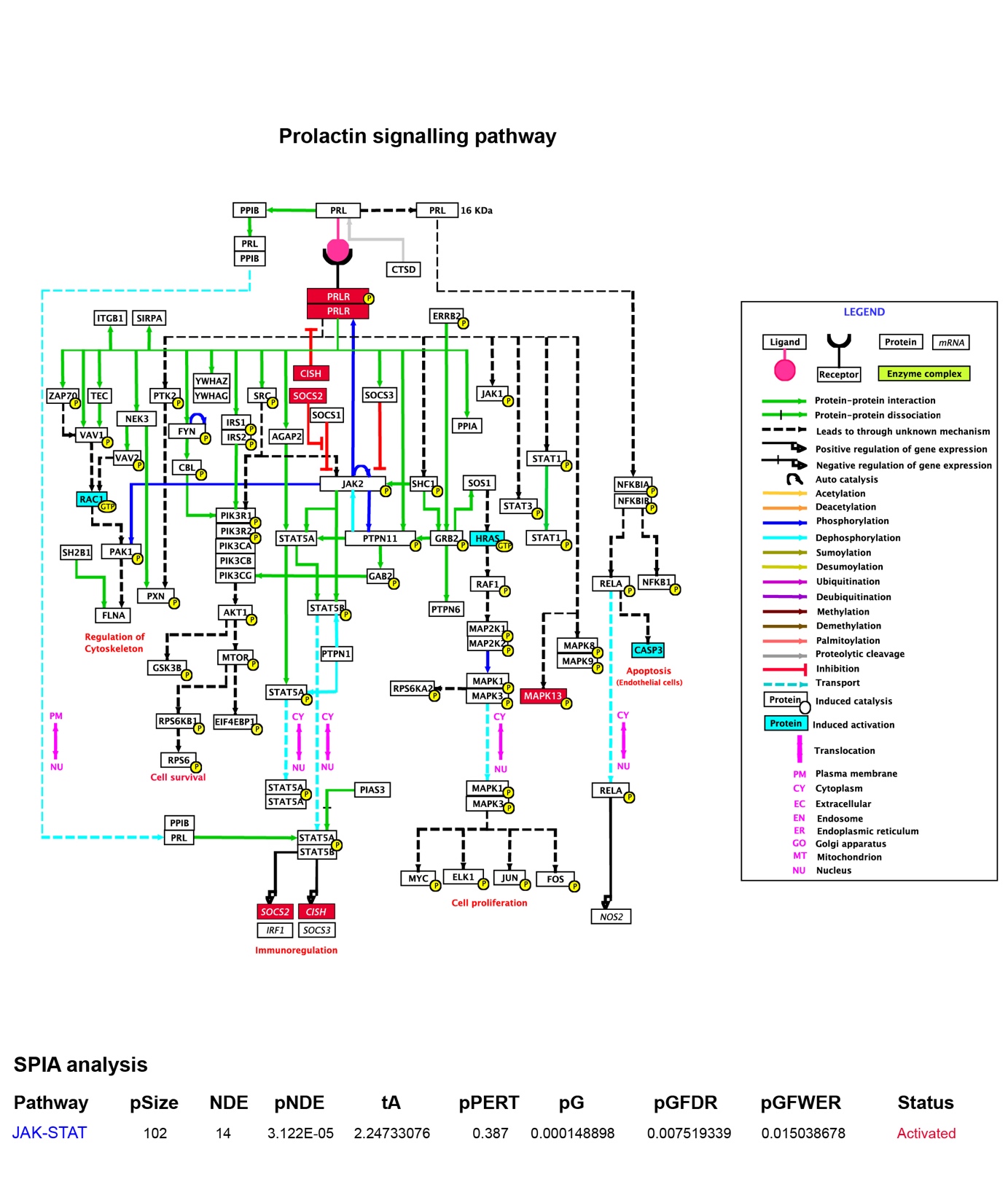


Figure S8. The PRL signaling pathway. Genes upregulated in the female mouse adrenal gland compared to male at P12 weeks are highlighted in red. SPIA analysis showed that the PRL-regulated JAK-STAT pathway is significantly activated in female adrenals compared to male at P12 weeks of age. SPIA parameters: pSize, number of genes in the pathway; NDE, number of differentially expressed genes in the pathway; pNDE, hypergeometric probability of observing NDE genes in the pathway by chance; tA, observed value of the perturbation score; pPERT, bootstrap probability associated to tA; pG, combined probability of pNDE and pPERT; pGFDR, adjusted pG using False Discovery Rate correction; pGFWER, adjusted pG using Family Wise Error Rate (Bonferrroni); Status, Inhibbition/Activation according to the negative/positive sign of tA.


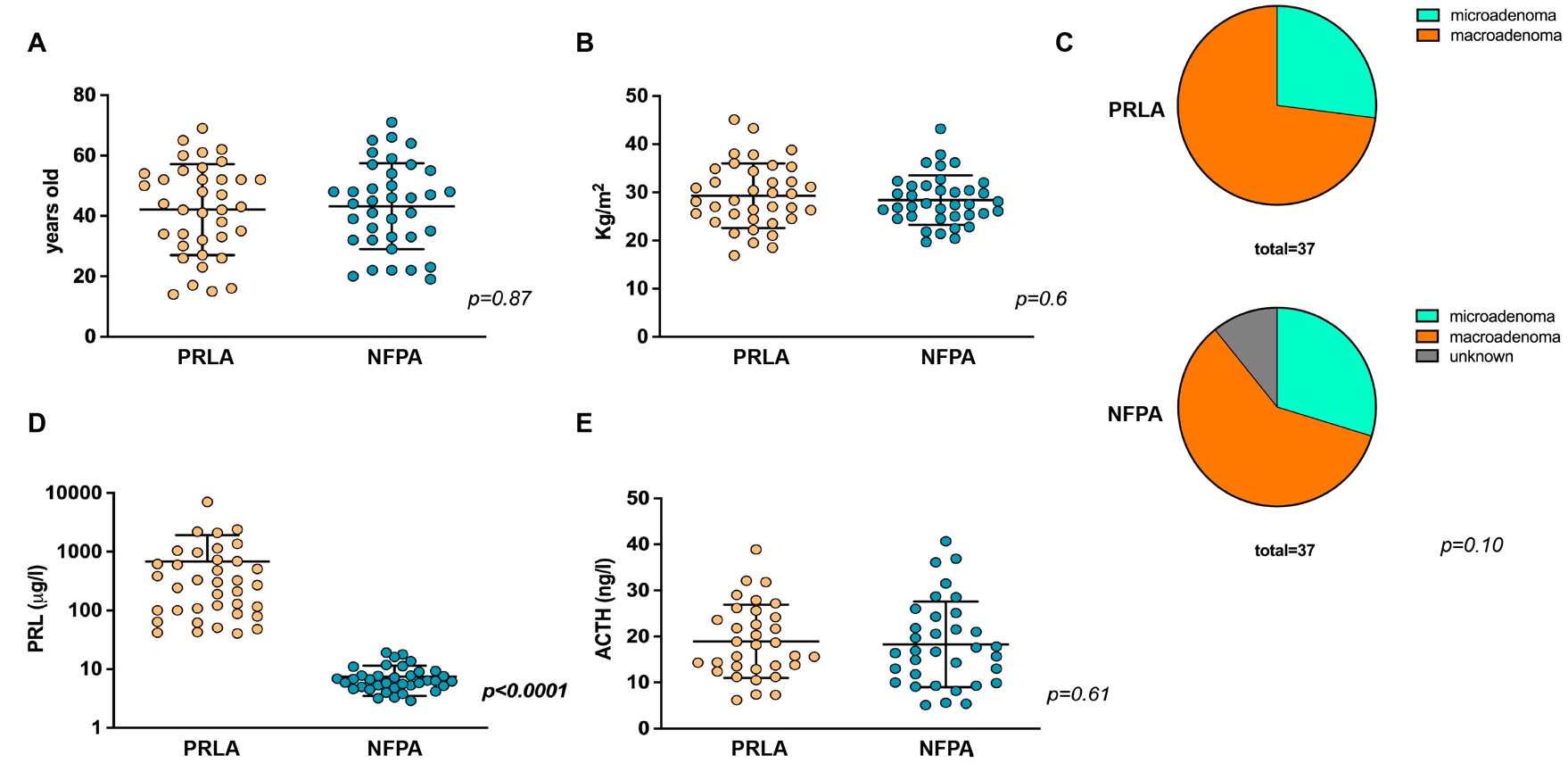


Figure S9. Clinical and biochemical parameters of patients with PRLA and NFPA investigated in this study. (A) Age at the time of hormonal measurement; (B) Body mass index; (C) Tumour size: microadenoma, <1 cm; macroadenoma, ≥1 cm; (D) Plasma PRL levels and (E) ACTH levels in patients with PRLA and NFPA, respectively. *n*=37 for each group. The Mann-Whitney test was used to assess the statistical significance of differences between groups in panels A, B, D and E and the χ^2^ test in panel C. Statistical significance values are reported in each panel.

**Supplementary Tables**

**Table S1**. DEG in the mouse adrenal gland according to age (sex-matched samples)

**Table S2.** DEG in the mouse adrenal gland according to sex

**Table S3**. Gene Ontology classification of mouse adrenal DEG according to age

**Table S4.** Gene Ontology classification of mouse adrenal DEG according to sex

**Table S5**. Differentially expressed lncRNAs

**Table S6**. Sex-dependent DEG in adrenal cell populations

**Table S7**. Mouse adrenal SEC and TEC at each age and in both sexes

**Table S8**. Gene Ontology classification of genes associated to adrenal gland SEC

**Table S9**. Association of human adrenal SEC with genes involved in blood pressure regulation

**Table S10**. *KCNK3* upstream SNPs associated with blood pressure traits

**Table S11.** Sex-dependent DEG in the adult rodent adrenal gland

**Table S12.** Clinical and biochemical parameters in patients with PRLA and NFPA

**Table S13**. RNA-seq data

**Table S14**. ChIP-seq data
