# Supplementary material for: Integrative genomic analysis reveals a conserved role for prolactin signalling in the regulation of adrenal function
Source: Clin Transl Med. 2021 Nov 8;11(11):e630. doi: 10.1002/ctm2.630 (PMC8574957; doi:10.1002/ctm2.630)
Supplement: Supplementary file 13 — Supporting information [file CTM2-11-e630-s015.docx]

**Table S12. Clinical and biochemical parameters in patients with PRLA and NFPA.**

| **Parameters** | **PRLA** | **NFPA** | ***p*, χ^2^** |
| --- | --- | --- | --- |
| n | 37 | 37 | - |
| F:M | 15:22 | 15:22 | - |
| Age - years | 47 (17-74) | 49 (20-72) | *0.52* |
| BMI - kg/m^2^ | 28.3 (16.9-45.1) | 27.5 (19.7-43.2) | *0.61* |
| Tumor size:  Microadenoma (<1 cm)  Macroadenoma (≥1 cm)  Unknown | 10 (27%)  27 (73%)  0 (0%) | 11 (29.7%)  22 (59.5%)  4 (10.8%) | *0.10*, 4.56 |
| ***Symptoms:***  Galactorrhea/gynecomastia  Symptoms of hypogonadism  Headache  Visual impairment | 8 (21.6%)  19 (51.4%)  15 (40.5%)  6 (16.2%) | 0 (0%)  7 (18.9%)  13 (35.1%)  3 (9.6%) | ***0.003***, 8.97  ***0.003***, 8.54  *0.63*, 0.23  *0.29*, 1.14 |
| ***Medical treatment***:  Dopamine agonists  Levothyroxine  Replacement with sex hormones  Antihypertensives | 16 (43.2%)  10 (27%)  8 (21.6%)  8 (21.6%) | 0 (0%)  10 (27%)  5 (13.5%)  9 (24.3%) | ***<0.001***, 20.41  *1*, 0  *0.36*, 0.84  *0.78*, 0.08 |
| ***Hormone measurements by Immulite***:  PRL -μg/l  ACTH -ng/l  DHEAS-μg/dl  Cortisol  LH -IU/l  FSH-IU/l  TSH* -mIU/l  FT4* -pmol/l  IGF-1-μg/l | 270.0 (40.7-7042.0)  18.2 (6.2-38.9)  184.5 (24.4-696.0)  9.6 (3.4-26.4)  1.6 (0.1-13.1)  2.6 (0.1-32.8)  1.8 (0.7-3.5)  12.1 (8.5-15.7)  123.0 (53.0-245.0) | 6.3 (2.9-19.1)  16.8 (5.1-40.7)  96.2 (15.0-316.0)  10.5 (1.1-19.5)  3.4 (0.1-25.1)  4.0 (0.1-98.9)  1.2 (0.5-2.3)  13.5 (4.5-18.9)  116.0 (25.0-309.0) | ***<0.0001***  *0.62*  ***0.029***  *0.60*  ***0.001***  ***0.004***  ***0.02***  ***0.005***  *0.93* |
| ***Hormone measurements by LC-MS/MS:***  Cortisol -μg/l  basal  after ACTH  Aldosterone* -ng/l  basal  after ACTH  Cortisone -μg/l  basal  after ACTH  Corticosterone basal -μg/l  basal  after ACTH  11-deoxycortisol -μg/l  basal  after ACTH  Testosterone* -μg/l  basal  after ACTH  Androstenedione -μg/l  basal  after ACTH  DHEA -μg/l  basal  after ACTH  17-OH-Progesterone -μg/l  basal  after ACTH | 10.4 (3.3-31.5)  27.0 (15.3-46.9)  50.6 (21.6-266.0)  115.0 (38.0-405.0)  22.6 (9.3-35.5)  19.9 (13.9-30.3)  1.5 (0.4-6.6)  27.0 (5.3-47.0)  0.2 (0.1-0.5)  1.2 (0.4-3.1)  0.8 (0.1-4.6)  0.9 (0.1-4.4)  0.7 (0.2-1.6)  1.5 (0.5-3.6)  2.8 (2.1-7.5)  9.1 (1.2-19.3)  0.2 (0.1-1.2)  1.3 (0.4-5.4) | 11.8 (0.9-30.0)  27.7 (9.21-51.3)  49.2 (21.7-231.0)  157.5 (28.1-520.0)  20.0 (1.5-32.6)  20.3 (9.4-26.2)  2.0 (0.3-8.9)  25.7 (5.9-55.8)  0.2 (0.1-0.8)  1.3 (0.4-3.9)  1.8 (0.1-6.9)  1.7 (0.1-7.2)  0.6 (0.1-2.0)  1.1 (0.3-2.5)  2.6 (0.4-8.2)  6.6 (1.0-20.4)  0.4 (0.1-2.4)  1.4 (0.8-7.1) | *0.64*  *0.75*  *0.89*  *0.34*  *0.65*  *0.85*  *0.45*  *0.57*  *0.16*  *0.45*  *0.13*  *0.27*  *0.18*  ***0.02***  *0.24*  *0.06*  ***0.009***  *0.11* |

Continuous variables are reported as median (range), whereas categorical variables are reported as numbers (percentages). Statistical analysis was performed by t-test or Mann-Whitney test or chi-square test (χ^2^), as appropriate. A *p* value in bold type indicates a significant difference (*p<0.05*). Abbreviation: ACTH, adrenocorticotropic hormone; after ACTH, 1 hour after 250 µg synthetic ACTH; BMI, body mass index; DHEA, dehydroepiandrosterone; DHEAS, dehydroepiandrosterone sulfate; F, female; FSH, follicle-stimulating hormone; FT4, free thyroxine; IGF-1, insulin-like growth factor-1; LC-MS/MS, liquid chromatography-tandem mass spectrometry; LH, luteinizing hormone; M, male; NFPA, non-functioning pituitary adenoma; PRLA, prolactin-secreting adenoma; TSH, thyroid stimulating hormone.

*For the final analyses, only patients without hormone supplementation therapies or medicaments interfering with the hormonal levels were evaluated: for TSH and FT4, 27 PRLA-patients and 27 NFPA-patients; for aldosterone, 29 PRLA-patients and 28 NFPA-patients; for testosterone, 35 PRLA-patients and 36 NFPA-patients.
